# Comparison of Long-Term Postoperative Outcomes of the Subtypes of Chronic Rhinosinusitis with Nasal Polyps

**DOI:** 10.3390/jcm13061699

**Published:** 2024-03-15

**Authors:** Sang-Min Lee, Shin-Hyuk Yoo, Ji-Hun Mo

**Affiliations:** Department of Otorhinolaryngology, College of Medicine, Dankook University, 119 Dandae-ro, Dongnam-gu, Cheonan 31116, Chungcheongnam-do, Republic of Korea; sangmin4865@dkuh.co.kr

**Keywords:** chronic rhinosinusitis, nasal polyps, subtype, disease control status, health-related quality of life, surgery

## Abstract

(1) **Background:** Chronic rhinosinusitis with nasal polyps (CRSwNP) is a chronic inflammatory condition that significantly impacts the health-related quality of life (HRQOL) of patients. This study aims to investigate the disparities in preoperative examination findings, postoperative HRQOL, and disease control status based on CRSwNP subtypes. (2) **Methods:** A retrospective analysis was conducted on 202 patients who underwent endoscopic sinus surgery for CRSwNP. The study assessed clinical characteristics, blood eosinophil and immunoglobulin E (IgE) levels, modified Lund–Kennedy and Lund–Mackay scores, and Japanese Epidemiological Survey of Refractory Eosinophilic Chronic Rhinosinusitis (JESREC) scores. HRQOL was evaluated using the Sino-nasal Outcome Test (SNOT-22) scores, and disease control status was assessed based on the European Position Paper on Rhinosinusitis and Nasal Polyps 2020 guidelines. (3) **Results:** Of the 202 patients, Eosinophilic CRSwNP patients exhibited significantly higher preoperative peripheral blood eosinophil ratios and IgE levels, and JESREC scores (*p* < 0.05). Two years postoperatively, patients in the non-eosinophilic group showed significantly improved SNOT-22 scores compared to preoperative scores (*p* = 0.007). Notably, the proportion of patients with uncontrolled disease was significantly higher in the eosinophilic group (*p* = 0.035). Logistic regression analyses identified preoperative SNOT-22 scores and eosinophilic CRSwNP subtype as influential factors on disease control status (*p* < 0.05). (4) **Conclusions:** Patients with more severe preoperative symptoms and eosinophilic CRSwNP demonstrated poorer long-term treatment outcomes.

## 1. Introduction

Chronic rhinosinusitis with nasal polyps (CRSwNP) is a chronic inflammatory disorder characterized by the presence of nasal polyps, persistent rhinosinusitis symptoms lasting more than 12 weeks, and sinusitis detected on sinus computed tomography (CT) scans [1]. CRSwNP often leads to a significant reduction in health-related quality of life (HRQOL) due to bothersome symptoms, including nasal congestion, posterior nasal drip, reduced sense of smell, facial pain, and sleep disturbances [2,3]. Although the exact pathophysiology of CRSwNP is not fully understood, it is known to be characterized by persistent mucosal inflammation brought on by either excessive or inappropriate immune responses to foreign substances, and there have also been studies associating CRSwNP to an autoimmune response [1,4].

CRSwNP can be classified into two main subtypes based on the presence of eosinophils in either tissue or blood: eosinophilic CRSwNP and non-eosinophilic CRSwNP. These subtypes exhibit distinct pathophysiological characteristics. In Caucasians, eosinophilic nasal polyps are predominant, and are associated with type 2 inflammation, often co-occurring with other type 2 inflammatory conditions like asthma [5,6,7]. In contrast, among East Asian patients with CRSwNP, 40–60% present with non-eosinophilic polyps driven by non-type 2 inflammation, and the prevalence of coexisting type 2 inflammatory diseases is lower [5,6,7]. Eosinophilic CRSwNP is known for its surgical challenges and frequent postoperative recurrence, whereas non-eosinophilic CRSwNP typically exhibits a more favorable postoperative prognosis [8,9]. Surprisingly, despite the differences in CRSwNP subtypes, previous studies have reported limited effects on symptoms and HRQOL [10], most of which have been conducted on Caucasian populations, leaving a gap in understanding the postoperative prognosis and HRQOL associated with CRSwNP subtypes among East Asian patients.

Hence, this study aims to elucidate disparities in preoperative clinical characteristics, postoperative HRQOL, and disease control status based on CRSwNP subtypes in a cohort of East Asian patients. We investigated how these differences influence long-term treatment outcomes, and the potential need for extended postoperative management and supplementary treatments. By exploring the nuances between eosinophilic and non-eosinophilic CRSwNP within the East Asian population, we aim to contribute valuable insights to the clinical management of this challenging chronic condition.

## 2. Materials and Methods

### 2.1. Study Population

This is retrospectively conducted study analyzed data from 202 patients who underwent bilateral endoscopic sinus surgery for the treatment of CRSwNP at a Dankook university hospital, a single tertiary center, between September 2014 and September 2018. The diagnosis of CRSwNP was established based on clinical symptoms, medical history, findings from nasal endoscopic examinations, and sinus CT imaging, in accordance with the EPOS 2020 guidelines [1]. The surgery was performed by two experienced rhinologists. Up to one month after surgery, follow-up was performed through an outpatient clinic at intervals of about 1–2 weeks, and after that, follow-up was performed every 2–3 months. In all patients, the same kind of nasal corticosteroid spray, oral antihistamines and antileukotrians, and sometimes oral steroids were used according to the postoperative patient’s symptoms and nasal endoscopy.

Patients with odontogenic sinusitis or neoplastic conditions such as inverted papilloma were excluded from the study, and patients who used biologics such as dupliumab after surgery were also excluded. The study was conducted according to the guidelines of the Declaration of Helsinki, and approved by the Institutional Review Board of Dankook University Hospital (IRB No. 2023-12-012, date of approval: 1 January 2024).

### 2.2. Data Collection

Patient demographics, clinical characteristics, and comorbidities including asthma, aspirin-exacerbated respiratory disease (AERD), and perennial allergies were recorded. Asthma and AERD diagnoses were confirmed by pulmonologists, while perennial allergies were determined using skin prick tests, ImmunoCAP, and the Multiple Allergen Simultaneous Test (MAST).

### 2.3. Blood Eosinophil and Immunoglobulin E Levels

Preoperatively, blood eosinophil percentages were determined through complete blood count (CBC) test, and total blood immunoglobulin E (IgE) levels were measured using the Pharmacia CAP assay (Uppsala, Sweden).

### 2.4. Historical Analyses and Classification of CRSwNP Subtype

CRSwNP can be divided into eosinophilic and non-eosinophilic types based on the number of eosinophils infiltrating the tissue [1,11]; however, there is no standardized definition for eosinophilic CRSwNP. In this study for evaluation of nasal histology, nasal polyp tissues were embedded in paraffin and sectioned with 4 μm thickness. The polyp slides were divided into three consecutive sections and stained with hematoxylin-eosin (HE). The highest cell density of three areas per slide was determined and observed at a 400-fold magnification. Following the criteria established in previous studies, the classification of ECRS and non-ECRS was based on the presence of 10% eosinophils per high-power field (HPF) [5,12].

### 2.5. Clinical Scoring

The Lund–Kennedy scoring system is the most frequently used method for evaluating a patient’s condition using endoscopic images [13]. Psaltis et al. [14] modified the Lund–Kennedy (MLK) scoring system using three items: polyp, oedema, and discharge. The MLK scoring system has shown high reliability and is correlated with the SNOT-22 score. The Lund–Mackay (LM) scoring system is the most acknowledged method for evaluating sinus CT images of patients with CRS [15,16].

The Japanese Epidemiological Survey of Refractory Eosinophilic Chronic Rhinosinusitis (JESREC) score was used to predict the subtype and prognosis of the patients with CRS. The JESREC score is based on the ethmoid/maxillary ratio on sinus CT images, peripheral blood eosinophil ratio, presence of bronchial asthma as a comorbidity, and non-steroidal anti-inflammatory drug intolerance [17]. Eosinophilic-type CRS is defined as a JESREC score of ≥11, and a higher score indicates greater disease severity, refractoriness to treatment, and disease recurrence rate.

To assess the severity of disease, MLK scores were assigned based on nasal endoscopic findings, while LM scores were employed to evaluate sinus CT imaging. Additionally, JESREC scores were calculated.

### 2.6. HRQOL Assessment

Sino-nasal Outcome Test-22 (SNOT-22) is widely used for evaluating the treatment outcome after surgery for CRS [18,19,20]. It can be largely divided into four domains: rhinologic (nasal obstruction, runny nose, cough, sneezing, loss of smell, posterior nasal drip), sleep disorder (difficulty in falling asleep, frequent wake up, lack of deep sleep waking up tired) psychological dysfunction (lack of concentration, anxiety, irritable, embarrassed, sad), and ear/facial symptoms (earfullness, ear pain, dizziness, facial tenderness or pain) [21]. The total score and the score of each domain reflect the overall disease burden and HRQOL status in patients with CRSwNP [18,19,20,21,22,23]. In this study, the HRQOL status was determined preoperatively and at the last follow-up date postoperatively using SNOT-22.

### 2.7. Disease Control Status

The postoperative patient condition can also be evaluated by assessing the disease control status, according to the EPOS 2020 guidelines, using the VAS scores. After evaluating the symptoms of rhinosinusitis, the control status can be divided into three categories according to the EPOS 2020 VAS scores: controlled, partially controlled, and uncontrolled. A strong correlation has been observed between the individual items measured using SNOT-22 and VAS scores [1,24]. In this study, the patient’s disease control status, according to the EPOS 2020 guidelines, was assessed at 1 year postoperative outpatient clinic visit through patient’s medical records and nasal endoscopy.

### 2.8. Statistical Analysis

All analyses were performed using SPSS (version 26.0; SSPS, Inc., IBM Company, Chicago, IL, USA) and GraphPad Prism version 8 (GraphPad Software, La Jolla, CA, USA). Independent and paired *t*-tests, Pearson’s chi-square test, and logistic regression analyses were performed to analyze the data. A *p*-value of <0.05 was considered statistically significant.

## 3. Results

### 3.1. Demographic and Clinical Characteristics according to CRSwNP Subtypes

**Figure 1:**
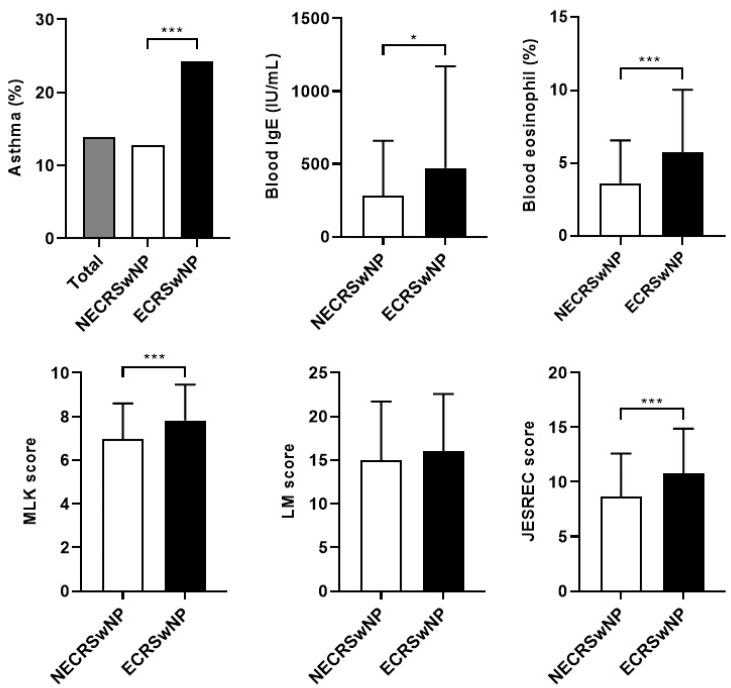
Difference between the results of the preoperative examinations of the two groups. NECRSwNP, non-eosinophilic subtype of chronic rhinosinusitis with nasal polyps; ECRSwNP, eosinophilic subtype of chronic rhinosinusitis with nasal polyps; IgE, immunoglobulin E; MLK, modified Lund–Kennedy; LM, Lund–Mackay; JESREC, Japanese Epidemiological Survey Of Refractory Eosinophilic Chronic Rhinosinusitis. *, *p* ≤ 0.05; ***, *p* ≤ 0.001.

Table 1 presents the demographic and clinical characteristics of the participants. Among the 202 patients with CRSwNP enrolled in this study, 128 (63.4%) and 74 (36.6%) patients were classified into the non-eosinophilic and eosinophilic CRSwNP, respectively. Of the total, 155 were male, and 47 were female, and there was no significant difference in gender ratio according to subtype. There was no significant difference by subtype in age, follow-up duration, and perennial allergy. Twenty-eight (13.9%) patients, comprising 10 patients (12.8%) from the non-eosinophilic group and 18 patients (24.3%) from the eosinophilic group, had asthma. The comorbidity of asthma was significantly higher in the eosinophilic group (*p* < 0.001). Eight patients (4.0%) from the eosinophilic group had AERD. On the other hand, there were no AERD patients in the non-eosinophilic group (*p* < 0.001). The preoperative blood test revealed that the mean blood IgE levels in the non-eosinophilic and eosinophilic groups were 279.5 ± 377.6 IU/mL and 472.1 ± 693.8 IU/mL, respectively, indicating that the IgE level in the eosinophilic group was significantly higher than that in the non-eosinophilic group (*p* = 0.012). The mean peripheral blood eosinophil ratio in the non-eosinophilic and eosinophilic groups were 3.6 ± 2.9% and 5.8 ± 4.2% (*p* < 0.001), respectively, indicating that the mean eosinophil ratio in the eosinophilic group was significantly higher than that in the non-eosinophilic group. The preoperative MLK score in the non-eosinophilic and eosinophilic groups were 6.95 ± 1.63 and 7.81 ± 1.64, (*p* < 0.001), respectively, indicating that the endoscopic score in the eosinophilic group was significantly higher than that in the non-eosinophilic group. The preoperative LM score in the non-eosinophilic and eosinophilic groups were 14.96 ± 6.55 and 16.07 ± 6.45, respectively, indicating that there was no significant difference between the two groups. The mean JESREC score in the non-eosinophilic and eosinophilic groups were 8.6 ± 4.0 points and 10.8 ± 4.1 points (*p* < 0.001), respectively, indicating that the mean JESREC score in the eosinophilic group was significantly higher than that in the non-eosinophilic group (Figure 1). The preoperative SNOT-22 score was 46.5 ± 23.8 points in the non-eosinophilic group and 49.6 ± 19.8 points in the eosinophilic group, showing a substantial reduction in HRQUL status in CRSwNP patients, with no significant difference between the two groups.

### 3.2. Annual Changes in the SNOT-22 Scores

**Figure 2:**
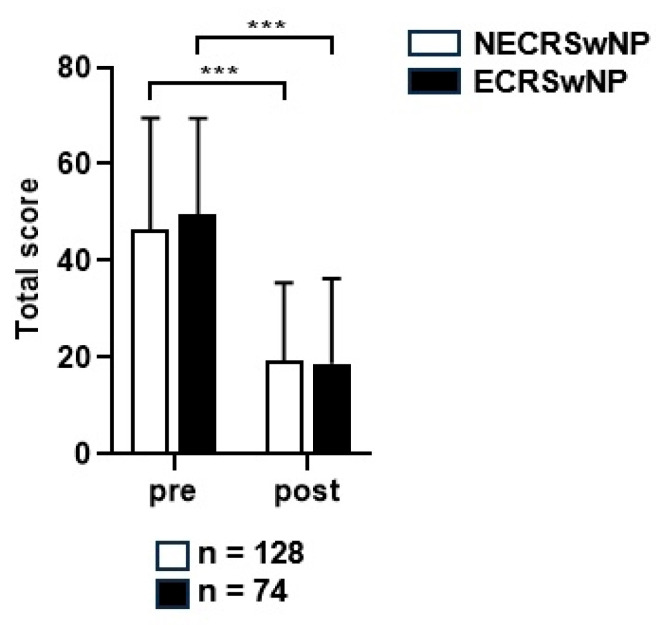
The preoperative and postoperative SNOT-22 scores of the two groups. SNOT-22, Sinonasal Outcome Test; NECRSwNP, non-eosinophilic subtype of chronic rhinosinusitis with nasal polyps; ECRSwNP, eosinophilic subtype of chronic rhinosinusitis with nasal polyps. ***, *p* ≤ 0.001.

**Figure 3:**
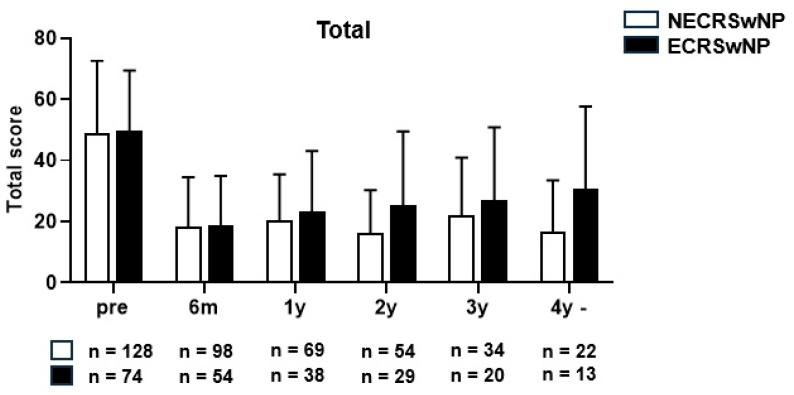
The mean SNOT-22 score of the patients with CRSwNP according to the years (y) after surgery (Total). SNOT-22, Sinonasal Outcome Test, NECRSwNP, non-eosinophilic subtype of chronic rhinosinusitis with nasal polyps; ECRSwNP, eosinophilic subtype of chronic rhinosinusitis with nasal polyps.

The average SNOT-22 score decreased from 46.5 ± 23.8 points preoperatively to 19.3 ± 15.3 points postoperatively in the non-eosinophilic group, and from 49.6 ± 19.8 points preoperatively to 18.7 ± 16.6 points postoperatively in the eosinophilic groups at the last follow-up date, indicating a significant decrease in both groups (*p* < 0.001) (Figure 2). An independent *t*-test was performed for all patients with follow-up data available by year to determine whether a significant difference in the mean SNOT-22 scores was present between the two patient groups. The mean SNOT-22 score tended to be lower in the non-eosinophilic group; however, there were no significant differences between the two groups at any time point (Figure 3).

**Figure 4:**
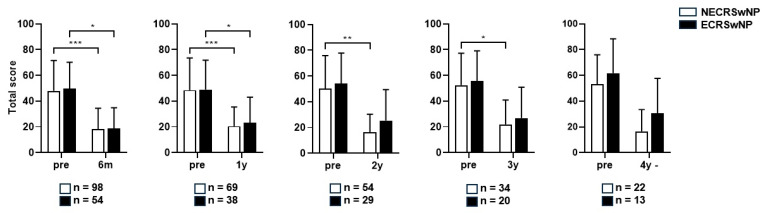
Comparison of the changes in the SNOT-22 score before and after surgery according to the subtype of CRSwNP (Total). SNOT-22, Sinonasal Outcome Test; NECRSwNP, non-eosinophilic subtype of chronic rhinosinusitis with nasal polyps; ECRSwNP, eosinophilic subtype of chronic rhinosinusitis with nasal polyps. *, *p* ≤ 0.05; **, *p* ≤ 0.01; ***, *p* ≤ 0.001.

An independent *t*-test was performed for the four domains of SNOT-22, and a significant difference was observed between the two groups in the rhinologic domain in the third postoperative year (*p* = 0.423). However, no significant differences were observed in the other domains (Figure S1). Paired *t*-tests revealed that the SNOT-22 scores remained significantly lower than the preoperative score until 1 year postoperatively in both groups. The SNOT-22 scores remained significantly lower in the non-eosinophilic group for 2–3 years postoperatively, but not in the eosinophilic group. The SNOT-22 score remained significantly lower in the non-eosinophilic group up to a year when both groups had significance (Figure 4).

**Figure 5:**
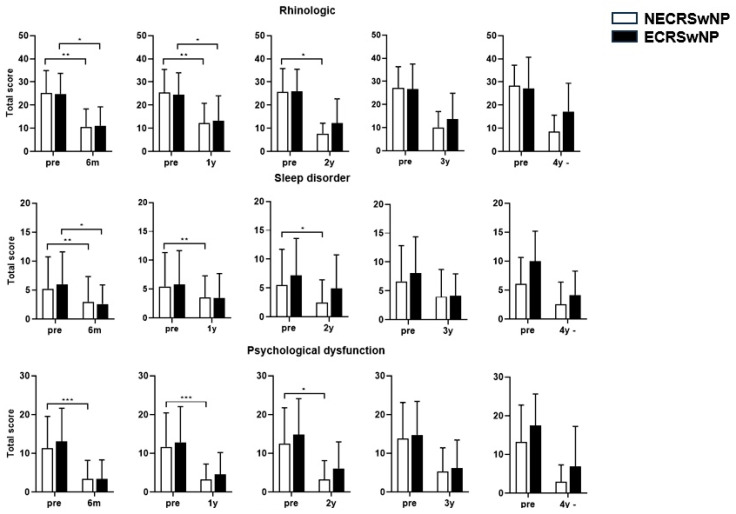

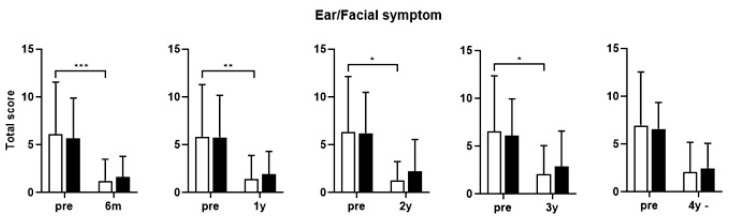
Comparison of the changes in the SNOT-22 score before and after surgery according to the subtype of CRSwNP (Domain). SNOT-22, Sinonasal Outcome Test; NECRSwNP, non-eosinophilic subtype of chronic rhinosinusitis with nasal polyps; ECRSwNP, eosinophilic subtype of chronic rhinosinusitis with nasal polyps. *, *p* ≤ 0.05; **, *p* ≤ 0.01; ***, *p* ≤ 0.001.

Paired *t*-tests performed for each domain of the SNOT-22 revealed that the score for the rhinologic domain remained significantly lower in the eosinophilic group 2 years postoperatively. Moreover, a difference was observed between the two groups in the sleep disorder domain from the first postoperative year. The difference began to appear at 6 months in the psychological symptom domain. A difference was also observed in the ear/facial symptom domain (Figure 5).

Thus, the total SNOT-22 score and the scores for its domains were not well-controlled in the eosinophilic group, indicating that the symptoms of rhinosinusitis were not well-controlled in the eosinophilic group compared with the non-eosinophilic group postoperatively.

### 3.3. Disease Control Status according to EPOS 2020 Guideline

**Figure 6:**
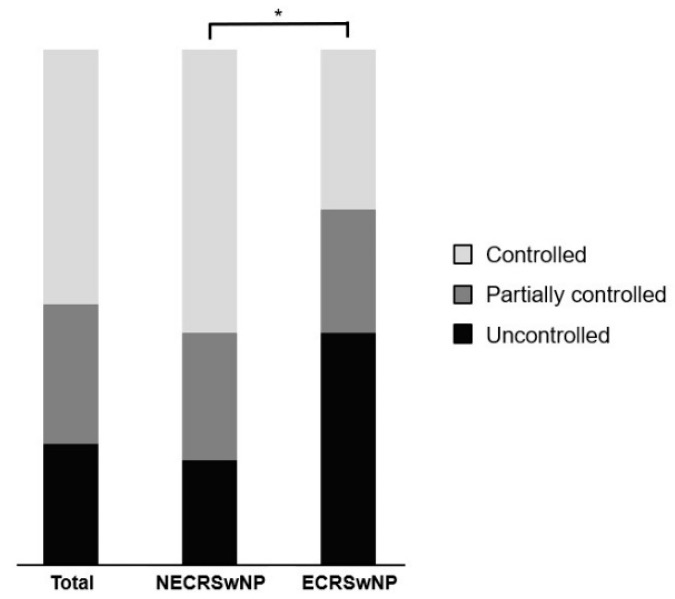
Disease control status according to the EPOS 2020 guideline in all patients and the patients with the eosinophilic and non-eosinophilic subtypes of CRSwNP. EPOS, European Position Paper on Rhinosinusitis and Nasal Polyps; NECRSwNP, non-eosinophilic subtype of chronic rhinosinusitis with nasal polyps; ECRSwNP, eosinophilic subtype of chronic rhinosinusitis with nasal polyps. *, *p* ≤ 0.05.

**Figure 7:**
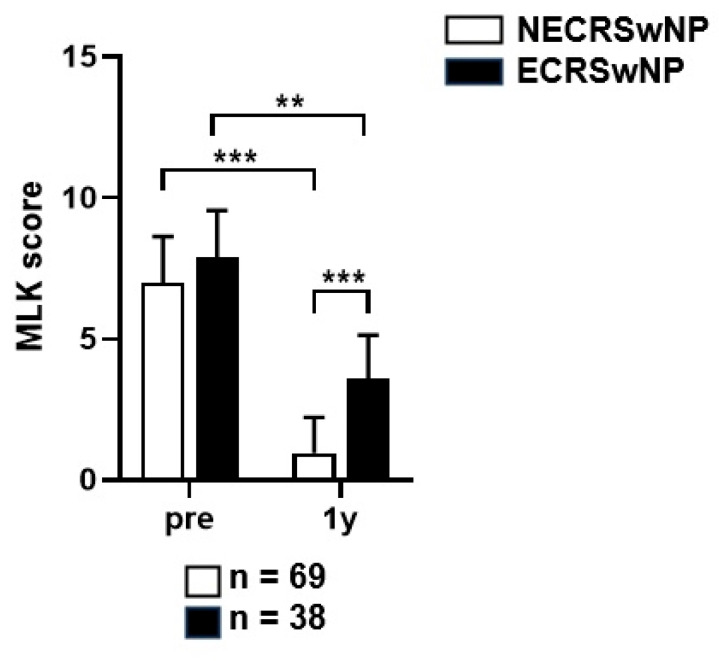
Comparison of the changes in the endoscopic score before and after a year of surgery according to the subtype of CRSwNP. MLK, Modified Lund–Kennedy; NECRSwNP, non-eosinophilic subtype of chronic rhinosinusitis with nasal polyps; ECRSwNP, eosinophilic subtype of chronic rhinosinusitis with nasal polyps. **, *p* ≤ 0.01; ***, *p* ≤ 0.001.

Among the 107 patients who visited the outpatient clinic over 1 years after surgery, 69 (64.5%) had non-eosinophilic CRSwNP and 38 (34.4%) had eosinophilic CRSwNP. Among all the patients, 25 (23.4%) were uncontrolled, 29 (37.1%) were partially controlled, and 53 (49.5%) were controlled. In the non-eosinophilic group, the disease status was uncontrolled, partially controlled, and controlled in 14 (20.3%), 17 (24.6%), and 38 (55.1%) patients, respectively. In the eosinophilic group, the disease status was uncontrolled, partially controlled, and controlled in 15 (39.5%), 28 (21.0%), and 15 (39.5%) patients, respectively (Figure 6). The proportion of patients with uncontrolled disease was higher in the eosinophilic group than that in the non-eosinophilic group, and the chi-square test showed a statistically significant difference between the two groups (*p* = 0.035). The MLK score was re-measured based on the nasal endoscopy for the evaluation of the disease control status. Paired *t*-tests showed that the MLK score at year 1 after surgery was 7.00 ± 1.62 preoperatively and 0.95 ± 1.25 postoperatively in the non-eosinophilic group, 7.89 ± 1.61 preoperativley and 3.85 ± 1.51 postoperatively in the eosinophilic group. The postoperative MLK score decreased significantly in both groups, but it was less so in the eosinophilic group (*p* < 0.001, *p* = 0.013), and the postoperative MLK score was also significantly higher in the eosinophilic group (*p* < 0.001) (Figure 7). Logistic regression analysis conducted to identify factors affecting the disease control status revealed that the preoperative SNOT-22 score and eosinophilic CRSwNP subtype had an impact on the disease control status (Table 2). Patients with higher SNOT-22 scores and those with eosinophilic CRSwNP were more likely to have an uncontrolled disease status postoperatively.

## 4. Discussion

This study investigated the differences in the results of the preoperative examinations, changes in the HRQOL, and the disease control status according to the subtype in patients with CRSwNP. Among the 202 patients included in this study, 128 (63.4%) and 74 (36.6%) had non-eosinophilic and eosinophilic CRSwNP, respectively, which was not significantly different from the rates previously reported in East Asian populations [6].

Asthma is a common comorbidity of CRSwNP. In Europe, asthma is a comorbidity in 20–60% of patients with CRSwNP [24]. In the present study, 13.9% of the patients had asthma, which was lower than that reported previously. However, 24.3% of the patients in the eosinophilic group had asthma as a comorbidity, which is consistent with the findings of previous reports, and was significantly higher than that in the non-eosinophilic group.

IgE plays a critical role in mediating inflammatory responses, particularly allergic reactions, mild immune responses to asthma or parasites, and type 2 inflammatory responses via the Th2 cells [25,26]. The blood IgE levels increase with the severity of CRSwNP [27]. Also, Type 2 inflammatory reactions lead to eosinophil differentiation, blood eosinophilia, and tissue infiltration of the eosinophils. Blood eosinophilia is positively correlated with the number of infiltrating eosinophils in the tissue, and is an important risk factor for uncontrolled CRSwNP [25,26,27,28,29,30]. In this study, preoperative blood tests revealed that the blood IgE levels and eosinophil ratios were significantly higher in the eosinophilic group (Figure 1), indicating type 2 inflammation, as reported in previous studies [25,26,27,28,29]. However, they did not appear to be significant factors affecting patient’s disease control status (Table 2). The preoperative JESREC score was also significantly higher in the eosinophilic group, which is consistent with the findings of previous reports [20].

The HRQOL status after endoscopic sinus surgery was assessed using SNOT-22, and it was found that the HRQOL in the eosinophilic group was lower than that in the non-eosinophilic group. This difference was evident in the second postoperative year. Although disease recurrence should be determined by comprehensively analyzing the symptoms, findings of endoscopic examinations, and radiologic images, these results suggest that the disease may relapse or poor surgical outcomes may be observed in patients with eosinophilic CRSwNP [8,9,31].

One of the key findings of this study was the disparity in disease control status between the two subtypes according to the EPOS 2020 guidelines. Evaluation of the disease control status using the VAS item score system according to the EPOS 2020 guidelines revealed that the proportion of patients with uncontrolled disease was significantly higher in the eosinophilic group. Logistic regression analysis identified the preoperative SNOT-22 score and eosinophilic CRSwNP subtype as significant factors influencing disease control status. Patients with higher preoperative SNOT-22 scores and those with eosinophilic CRSwNP were more likely to have uncontrolled disease postoperatively. A meta-analysis reported a significant decrease in the postoperative SNOT-22 scores in patients with higher preoperative SNOT-22 scores [32]. However, higher preoperative SNOT-22 scores were found to inhibit disease control in patients. SNOT-22 reflects the subjective symptoms of the patient, and patients with high SNOT-22 scores tend to be sensitive to their own symptoms. Thus, patients with high preoperative SNOT-22 scores may be sensitive to minor residual symptoms, even if the symptoms resolve significantly after surgery. In the case of the EPOS 2020 VAS score, the disease was not considered to be fully controlled if any of the chronic rhinosinusitis symptoms persisted above a certain threshold. Thus, a higher preoperative SNOT-22 score can be considered an impediment to disease control.

The SNOT-22 and VAS scoring systems are widely used to evaluate the disease status of patients with CRSwNP before and after treatment. Although it is widely known that the postoperative prognosis of eosinophilic CRSwNP is worse than that of non-eosinophilic CRSwNP, few studies have evaluated the disease status postoperatively using the SNOT-22 and EPOS 2020 VAS scores in both patient groups. The present study evaluated the annual changes in the postoperative HRQOL status in both patient groups using SNOT-22.

One limitation of this study is the declining number of patients participating in follow-up assessments over time. Consequently, the study cohort included only 22 patients with non-eosinophilic CRSwNP and 13 patients with eosinophilic CRSwNP at the 4-year postoperative mark. Notably, the total SNOT-22 score and the scores within its four domains exhibited no significant differences between the two groups during the 3–4 year postoperative period. This suggests that the beneficial effects of surgery may wane after 3 years in both subgroups. Further studies with larger numbers of patients will need to be performed to confirm these results and to have more comprehensive analysis of the distinctions between these groups in the 3–4 year postoperative phase. Furthermore, it is essential to acknowledge that this study primarily focused on the clinical aspect, thereby overlooking the pathophysiological dimension. While numerous studies have endeavored to elucidate the pathophysiology of CRSwNP subtypes, the present study suggests that a more nuanced comprehension of CRSwNP may be attainable through follow-up investigations, which validate distinctions in histopathological characteristics or cytokine expression patterns even within the same subtype.

Nonetheless, this study holds substantial significance as it underscores the parallel clinical characteristics between East Asian patients with eosinophilic CRSwNP and their Caucasian counterparts. These similarities encompass the comorbidities of asthma and AERD, elevated blood eosinophil ratios, increased blood IgE levels, and an unfavorable postoperative prognosis. Consequently, it underscores the imperative of recognizing the disparities between these two subtypes when managing patients with CRSwNP, especially those with eosinophilic CRSwNP.

## 5. Conclusions

Patients with more severe preoperative symptoms and eosinophilic CRSwNP demonstrated poorer long-term treatment outcomes. This recognition may lead to the evaluation of additional therapeutic interventions, such as the administration of steroids or monoclonal antibodies like dupilumab and omalizumab, in conjunction with more frequent postoperative follow-up protocols.

## Figures and Tables

**Table 1:** Demographic and clinical characteristics of patients with CRSwNP according to subtype.

	Non-Eosinophilic (*n* = 128)	Eosinophilic (*n* = 74)	*p*
Gender			
Male, *n* (%)	98 (76.6)	57 (77)	
Female, *n* (%)	30 (23.4)	17 (23.0)	0.940
Age (y), Mean ± SD	45.7 ± 15.0	46.9 ± 12.5	0.549
Follow up duration (m), Mean ± SD	15.8 ± 19.5	15.1 ± 22.1	0.809
Allergy (Perennial), *n* (%)	82 (64.1)	54 (73.0)	0.284
Comorbid asthma, *n* (%)	10 (12.8)	18 (24.3)	<0.001
Comorbid AERD, *n* (%)	0 (0)	8 (10.8%)	<0.001
Smoker, *n* (%)	55 (43.0%)	28 (37.8%)	0.544
Blood IgE (IU/mL), Mean ± SD	279.5 ± 377.6	472.1 ± 693.8	0.012
Blood eosinophil ratio (%), Mean ± SD	3.6 ± 2.9	5.8 ± 4.2	<0.001
Pre-op MLK score, Mean ± SD	6.95 ± 1.63	7.81 ± 1.64	<0.001
Pre-op LM score, Mean ± SD	14.96 ± 6.55	16.07 ± 6.45	0.378
Pre-op JESREC score, Mean ± SD	8.6 ± 4.0	10.8 ± 4.1	<0.001
Pre-op SNOT-22 score, Mean ± SD	46.5 ± 23.8	49.6 ± 19.8	0.393

CRSwNP, chronic rhinosinusitis with nasal polyps; SD, standard deviation; AERD, aspirin exacerbated respiratory disease; IgE, immunoglobulin E; Pre-op, preoperative; JESREC, Japanese Epidemiological Survey of Refractory Eosinophilic Chronic Rhinosinusitis; LM, Lund–Mackay; MLK, Modified Lund–Kennedy; SNOT-22, Sinonasal Outcome Test-22.

**Table 2:** Logistic regression analysis of the factors affecting the control status according to EPOS 2020 guideline.

Factor	B	S.E.	Wald	*p*-Value	Exp (B)	95% CI for Exp (B)	
						Lower	Upper
Sex	−0.335	0.666	0.253	0.615	0.715	0.194	2.640
Age	−0.616	0.587	1.101	0.294	0.540	0.171	1.706
Allergy	−0.083	0.640	0.017	0.897	0.921	0.263	3.225
Asthma	0.304	0.792	0.147	0.701	1.355	0.287	6.395
AERD	−2.290	1.411	2.633	0.105	0.101	0.006	1.610
Smoking	−0.632	0.678	0.871	0.351	0.531	0.141	2.006
Pre-OP SNOT-22 score	−0.053	0.015	12.979	0.001 ^(a)^	1.054	1.024	1.085
Blood IgE	0.887	0.587	2.284	0.131	2.428	0.769	7.672
Blood eosinophil ratio	−0.923	0.878	1.106	0.293	0.397	0.071	2.219
Pre-OP MLK score	0.143	0.179	0.635	0.426	1.153	0.812	1.638
Pre-OP LM score	−0.025	0.051	0.241	0.624	0.975	0.883	1.078
Pre-OP JESREC score	1.191	0.899	1.755	0.185	3.289	0.565	19.152
Eosinophilic CRSwNP	1.361	0.598	5.178	0.023 ^(a)^	3.900	1.208	12.592

EPOS, European Position Paper on Rhinosinusitis and Nasal Polyps; CRSwNP, chronic rhinosinusitis with nasal polyps; AERD, aspirin exacerbated respiratory disease; SNOT-22, Sinonasal Outcome Test; Pre-OP, preoperative; CI, confidence interval; MLK, modified Lund–Kennedy; LM, Lund–Mackay; JESREC, Japanese Epidemiological Survey of Refractory Eosinophilic Chronic Rhinosinusitis. ^(a)^, *p* ≤ 0.05.

## Data Availability

Data sharing is not applicable to this article.

## Supplementary Materials

The following supporting information can be downloaded at: https://www.mdpi.com/article/10.3390/jcm13061699/s1. Figure S1. The mean SNOT-22 score of the patients with CRSwNP according to the year after surgery (Domain). SNOT-22, Sinonasal Outcome Test, NECRSwNP, non-eosinophilic subtype of chronic rhinosinusitis with nasal polyps; ECRSwNP, eosinophilic subtype of chronic rhinosinusitis with nasal polyps.
