# Supplementary material for: Comparison of Long-Term Postoperative Outcomes of the Subtypes of Chronic Rhinosinusitis with Nasal Polyps
Source: J Clin Med. 2024 Mar 15;13(6):1699. doi: 10.3390/jcm13061699 (PMC10971608; doi:10.3390/jcm13061699)

Figure S1. The mean SNOT-22 score of the patients with CRSwNP according to the year after surgery (Domain). SNOT-22, Sinonasal Outcome Test, NECRSwNP, non-eosinophilic subtype of chronic rhinosinusitis with nasal polyps; ECRSwNP, eosinophilic subtype of chronic rhinosinusitis with nasal polyps

*, $P \leq 0.05$

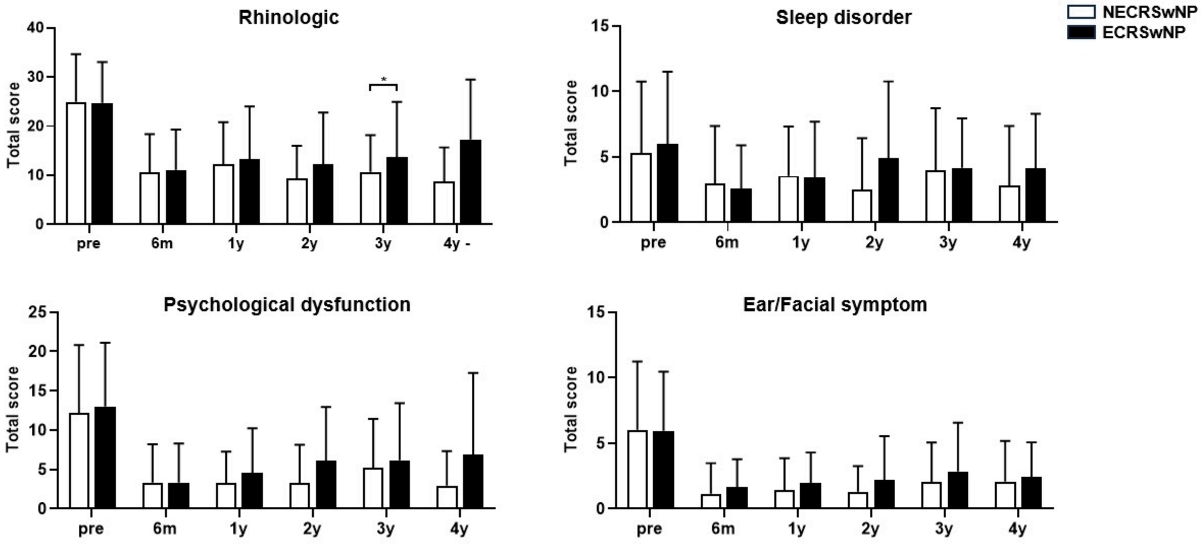

Supplement: Supplementary file 1 [file jcm-13-01699-s001.zip › jcm-2873202-supplementary.pdf]
